# Diagnostic difficulties in bipolar disorder type II

**DOI:** 10.1192/j.eurpsy.2023.1468

**Published:** 2023-07-19

**Authors:** L. Núñez, C. A. del Val, A. F. Merlos, E. X. G. Vivero, C. C. Carrascosa, M. H. Naval

**Affiliations:** Psychiatry, Instituto de Psiquiatria y Salud Mental, Hospital Clínico San Carlos, Madrid, Spain

## Abstract

**Introduction:**

Bipolar disorder is one of the top 10 medical causes of disability according to the WHO and despite this, its diagnosis can be delayed up to 10 years after the appearance of the first symptoms of the disease. A major reason for the difficult diagnosis is the challenge of differentiating bipolar disorder type II from unipolar depression and borderline personality disorder, especially in those patients with no clear history of hypomania.

**Objectives:**

To present a case report of a bipolar disorder undiagnosed for years to remark the importance of recognizing premorbid symptoms of the disease in order to implement an early intervention that potentially improves the prognosis of patients.

**Methods:**

We compiled the patient’s complete medical history and we carried out a non-systematic review of literature containing the key-words “bipolar disorder type II” and “diagnosis”.

**Results:**

We present the case of a 48-year-old woman going through a depressive episode, multiple suicide attempts and more than 10 admissions in the Acute Inpatient Psychiatric Unit. For 3 years, the evolution was torpid with a significant multidomain cognitive impairment in a previously functional patient. Different antidepressant treatments were tested, however they were not tolerated due to adverse effects such as anxiety, insomnia and nervousness. After considering multiple differential diagnoses, bipolar disorder type II was finally diagnosed. A hypomanic episode that took place after 3 sessions of electroconvulsive therapy during an admission for depression, allowed to guide the diagnosis and after the introduction of Lithium and Quetiapine as treatment, the patient experienced a complete remission of the symptoms.

**Conclusions:**

1. It is important to consider the differential diagnosis of bipolar disorder type II due to its impact on the patient´s life.
2. An early diagnosis improves the course and prognosis of the disease.
3. Patients resistant or intolerant to antidepressant treatment could have undiagnosed bipolar disorder.

**Disclosure of Interest:**

None Declared

